# Cardiovascular disturbances in COVID-19: an updated review of the pathophysiology and clinical evidence of cardiovascular damage induced by SARS-CoV-2

**DOI:** 10.1186/s12872-022-02534-8

**Published:** 2022-03-09

**Authors:** Ismaheel O. Lawal, Mankgopo M. Kgatle, Kgomotso Mokoala, Abubakar Farate, Mike M. Sathekge

**Affiliations:** 1 Department of Nuclear Medicine, University of Pretoria, Pretoria, 0001 South Africa; 2 Nuclear Medicine Research Infrastructure, Steve Biko Academic Hospital, Pretoria, South Africa; 3 Department of Radiology, University of Maiduguri, Maiduguri, Nigeria

**Keywords:** SARS-CoV-2, COVID-19, Cardiovascular disorders, Takotsubo cardiomyopathy, Thromboembolism, Positron emission tomography, Myocarditis

## Abstract

Severe acute respiratory coronavirus-2 (SARS-Co-2) is the causative agent of coronavirus disease-2019 (COVID-19). COVID-19 is a disease with highly variable phenotypes, being asymptomatic in most patients. In symptomatic patients, disease manifestation is variable, ranging from mild disease to severe and critical illness requiring treatment in the intensive care unit. The presence of underlying cardiovascular morbidities was identified early in the evolution of the disease to be a critical determinant of the severe disease phenotype. SARS-CoV-2, though a primarily respiratory virus, also causes severe damage to the cardiovascular system, contributing significantly to morbidity and mortality seen in COVID-19. Evidence on the impact of cardiovascular disorders in disease manifestation and outcome of treatment is rapidly emerging. The cardiovascular system expresses the angiotensin-converting enzyme-2, the receptor used by SARS-CoV-2 for binding, making it vulnerable to infection by the virus. Systemic perturbations including the so-called cytokine storm also impact on the normal functioning of the cardiovascular system. Imaging plays a prominent role not only in the detection of cardiovascular damage induced by SARS-CoV-2 infection but in the follow-up of patients’ clinical progress while on treatment and in identifying long-term sequelae of the disease.

## Background

Severe acute respiratory syndrome coronavirus 2 (SARS- CoV-2) is a single-stranded RNA virus that causes coronavirus disease 2019 (COVID-19). COVID-19 has caused significant disruption to the world order, including straining the health care systems even in industrialized and developed countries, crippling the world economy, and causing the loss of millions of lives.

SARS-CoV-2 was initially thought to unleash most of its cytotoxic effect on the respiratory tissues. Earlier reports from China and elsewhere showed that a history of cardiovascular disorders including cerebrovascular disease, hypertension, ischaemic heart disease, and heart failure was associated with an unfavorable outcome of COVID-19 [1–5]. Angiotensin-converting enzyme-2 (ACE-2), the cellular target used by SARS-CoV-2 to infect the cells, has also been shown to be expressed in cardiac myocytes [6]. Transmembrane serine protease 2 (TMPRSS2), the enzyme that primes SARS-CoV-2 spike protein facilitating its binding to ACE-2 for cellular entry, and other spike protein proteases including cathepsin L and furin (also known as PACE, paired basic amino acid cleaving enzyme) are also known to be expressed in cardiac myocytes [6, 7]. The findings of a higher rate of severe disease, hospitalization, and mortality among patients with SARS-CoV-2 infection and the confirmation of a high expression of ACE-2 in the cardiac tissue have fuelled the interest in the routine assessment of cardiac damage in patients evaluated for COVID-19.

Excellent review articles have been published discussing the cardiovascular changes in COVID-19. Until its first discovery in December 2019 in Wuhan, China, little was known about the impact of human infection with SARS-CoV-2. Since then, much work has been published elucidating the pathophysiology of the manifestations of this dreaded infection in the different organ-systems in human infection with SARS-CoV-2. This has made evidence available on this subject to be quite dynamic and evolving by the day. In this article, we aim to discuss the clinical assessment of patients with COVID-19 to identify cardiovascular manifestations of the disease. We also aim to describe the mechanisms behind the damage induced by SARS-CoV-2 based on the currently available evidence in the literature and conclude by providing insights on future directions for patients’ management.

We searched major databases including PUBMED, EMBASE, Cochrane database, and Scopus for relevant articles for discussion in this narrative review. The reference lists of identified articles were also searched for relevant articles.

## Cardiac injuries in COVID-19: the mechanistic theories

**Fig. 1:**
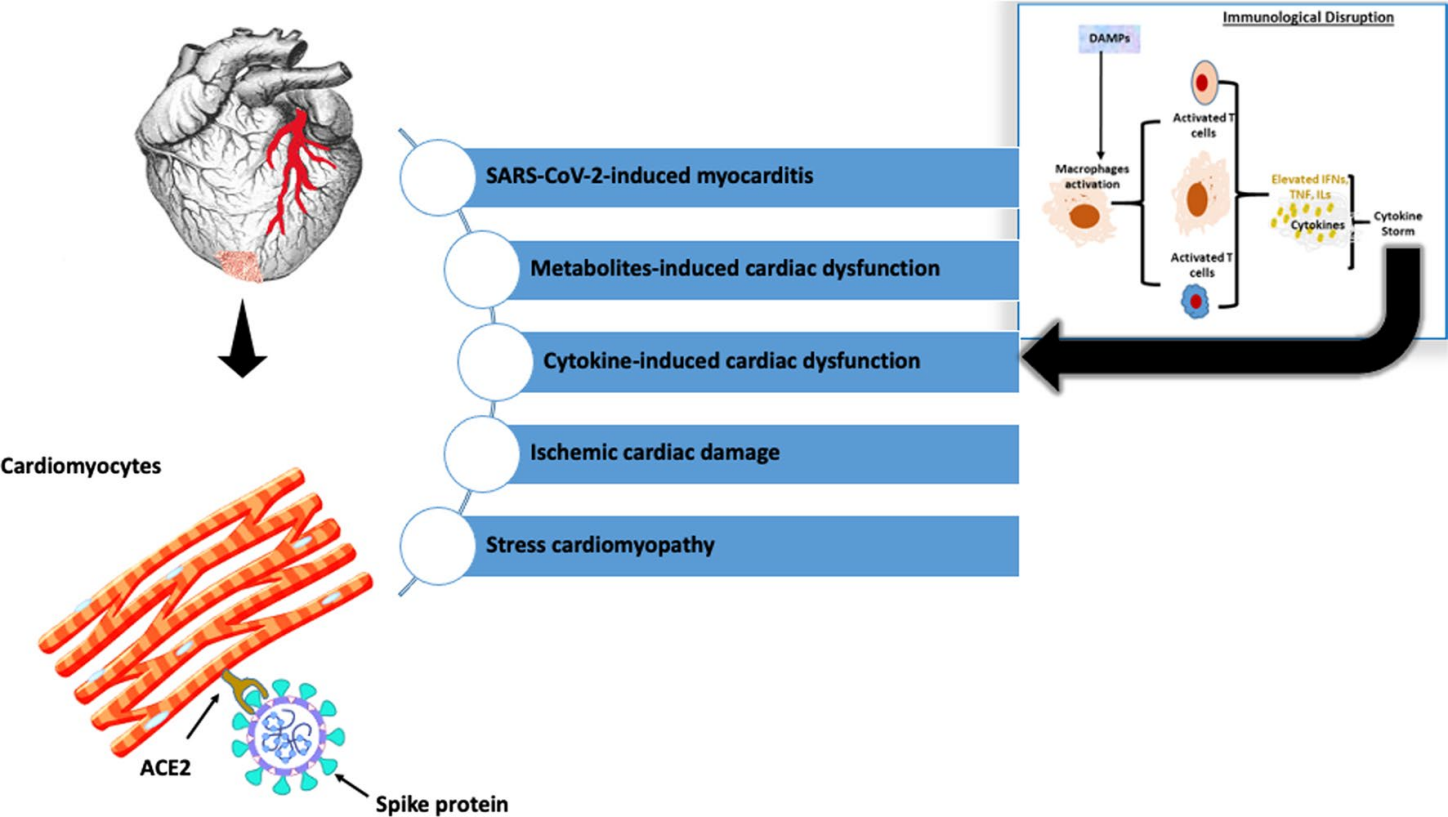
Schema showing the currently proposed mechanistic theories for the cardiac damage seen in COVID-19. Damage-associated molecular patterns (DAMPs) are molecules released in response to tissue damage. DAMPs cause an indiscriminate activation of the innate immune system propagating the heightened inflammatory response characteristic of severe COVID that contribute to myocardial dysfunction

The pathophysiological drivers of the heart changes induced by SARS-CoV-2 infection need to be understood to guide therapy development and for prognostication. Several mechanistic theories have been put forward to explain the pathophysiology of the cardiac damage induced by SARS-CoV-2 (Fig. 1). Demonstration of ACE-2 and the serine proteases necessary for spike protein priming in the heart led to the early suggestion that SAR-CoV-2 infection of the myocardium leading to viral-induced myocarditis was the cause of morphologic and functional derangements seen in COVID-19. An Italian autopsy series that reported myocarditis in 55% of patients who died of COVID-19 also supported this early theory [8]. In patients, the diagnosis of myocarditis is established based on histological findings on endomyocardial biopsy (EMB) specimens. EMB is a highly invasive procedure fraught with serious complications. EMB is, therefore, only indicated in patients in whom there is a strong reason to suggest that the procedure will meaningfully influence treatment decisions [9]. Postmortem examination provides an alternative method for a retrospective diagnosis of myocarditis in patients who succumbed to their disease. Myocarditis is best defined based on the immunohistochemical findings of abnormal inflammatory infiltrates (≥ 14 leucocytes/mm^2^ including up to 4 monocytes/mm^2^ and ≥ 7 CD3-positive T-lymphocytes) and non-ischaemic myocardial necrosis and degeneration [10]. Thorough reviews of the autopsy series published to date have demonstrated immunohistochemical findings that are convincing for SARS-CoV-2-induced viral myocarditis in only a few COVID-19 patients [11, 12]. This suggests that direct SARS-CoV-2-induced myocardial damage is not a prominent cause of the myocardial injury seen in COVID-19 patients.

Other plausible mechanisms of myocardial injury in COVID-19 have been put forth. SARS-CoV-2 infection is associated with an intense host inflammatory response accompanied by an excessive and uncontrollable release of cytokines, causing cytokine release syndrome (CRS) or cytokine storm. The serum levels of both proinflammatory cytokines like interleukin (IL)-1β, IL-6, and interferon-γ and T-helper-2 (Th2) anti-inflammatory cytokines like IL-4 and IL-10 are markedly elevated, with their levels corresponding to disease severity [13, 14]. IL-6 is critical as the major driver of CRS as it stimulates the production of other cytokines [15]. In addition, IL-6 promotes vascular leakage and interstitial edema [15]. In the myocardium, IL-6 impairs papillary muscle contraction and myocardial contractility [16]. The serum levels of IL-6 in particular, and other inflammatory biomarkers in general, track with the serum levels of cardiac enzymes suggesting an association between a rise in inflammatory biomarkers and cardiac damage [17].

Viral illnesses are known to increase the risk of plaque rupture and thrombus formation through the systemic inflammatory response they induce [18, 19]. The intense systemic inflammatory response accompanying SARS-CoV-2 infection may predispose to plaque rupture in the coronary vasculature causing ischaemic myocardial damage. Evidence is still lacking at present to support the contribution of plaque rupture to myocardial injury in COVID-19 patients [20]. Another factor that may predispose to myocardial ischaemic damage is the imbalance between increased myocardial oxygen demand induced by an unregulated systemic inflammatory response and the reduced oxygen supply to the myocardium caused by acute respiratory distress syndrome (ARDS). This imbalance between demand and supply of oxygen is aggravated by metabolic acidosis, electrolyte imbalance, and the dysregulation of the neurohormonal system [21].

Stress cardiomyopathy (Takotsubo cardiomyopathy) is a non-ischaemic cardiac dysfunction usually precipitated by emotional and physical stress. Conditions that trigger stress cardiomyopathy, including increased sympathetic discharge, microcirculatory dysfunction, proinflammatory state, and vasospasm, are known to be prevalent in COVID-19 patients [22]. Stress cardiomyopathy is a diagnosis of exclusion requiring the exclusion of all other potential causes of cardiac failure before its diagnosis can be established. Due to the significant challenges associated with a comprehensive cardiac assessment of severely ill COVID-19 patients, the reports on stress cardiomyopathy as a contributor to cardiac dysfunction in COVID-19 are limited to small series and case reports [23–25].

## Cardiac injury in COVID-19: the clinical evidence

### Biochemical evidence of cardiac damage in COVID-19

In the clinical setting, cardiac myocyte damage is assessed by measuring serum levels of cardiac enzymes such as troponins and myocardial-derive creatinine kinase and myocardial imaging. Measurement of serum levels of cardiac enzymes as a marker of cardiac damage at baseline and serially through the course of the disease is an easier and most used biomarker to track the dynamics of cardiac injury in COVID-19 patients. In the single-center retrospective observational study by Guo et al. that assessed the trajectory of cardiac troponin T (TnT) levels in 144 hospitalized patients who did not have cardiac damage at admission, patients who developed elevated TnT during hospitalization were more likely to be older and have co-morbid conditions such as hypertension, coronary artery disease, cardiomyopathy, among others [26]. TnT levels increased significantly during hospitalization among patients who died from COVID-19, while serum levels stayed relatively unchanged in patients who recovered from the disease [26]. These findings show that cardiac damage, as evident by raised serum cardiac enzyme levels, is more prominent in older patients and in individuals with comorbidities, and its presence is associated with higher mortality. Other studies have also reported a higher rate of mechanical ventilation, intensive care unit admission, and mortality in COVID-19 patients with elevated serum cardiac enzyme levels [27, 28]. In a meta-analysis of studies reporting cardiac damage in patients with COVID-19, Huang et al. reported a cardiac injury prevalence of 19% (95% confidence interval, CI: 15–22%) in unselected patient population, a prevalence of 36% (95% CI: 25–47%) in patients with severe COVID-19 and a prevalence of 48% (95% CI: 30–66%) in non-survivors [29].

### Imaging cardiac injury in COVID-19

**Fig. 2:**
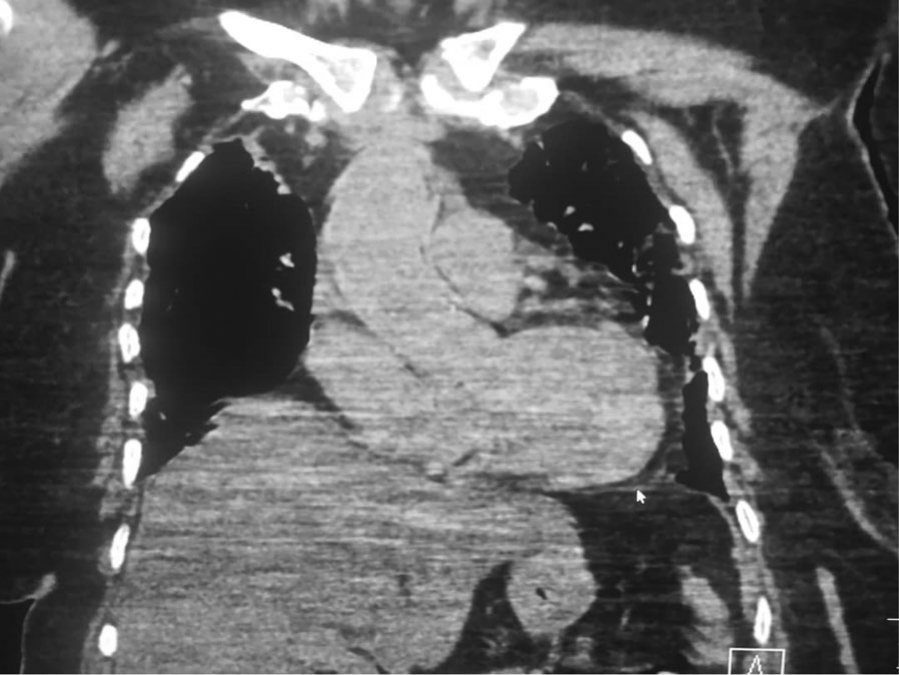
Coronal reformatted CT of the chest (mediastinal window) showing cardiomegaly with elevation of the cardiac apex (arrow), suggesting right ventricular dilatation

Cardiac damage seen in COVID-19 has been assessed with imaging [30, 31]. Echocardiography is a cheap and readily available imaging modality that does not utilize ionizing radiation. It can be used to image cardiac morphology and function at the bedside. New-onset cardiac failure and acute decompensation in patients known with cardiac failure are common in COVID-19 patients. Echocardiography can be useful in defining the ventricular function and wall motion abnormalities in COVID-19 patients who develop an acute cardiovascular event or hemodynamic instability [32]. There is an inverse correlation between left ventricular ejection fraction and TnT level [32]. The lungs are the primary organs affected by COVID-19 in which the predominant lung parenchymal abnormality especially in the early phase of the infection are bilateral ground-glass opacities. ARDS developing on the background of SARS-CoV-2-induced alveolar damage, therefore, puts a lot of strains on the right ventricle, which may lead to right ventricular dilatation and failure (Fig. 2). Echocardiography is technically demanding in COVID-19 patients due to the severity of the disease in patients in whom this imaging modality may be indicated and the risk of exposure of the operator to SARS-CoV-2 infection [33]. Compared with transoesophageal echocardiography, transthoracic echocardiography is non-invasive, may be easier to perform in the sick COVID-19 patients, and is particularly useful to assess right ventricular dilatation and function [34]. In a recent systematic review of studies evaluating echocardiographic findings in COVID-19 patients, Hassani et al. reported that right ventricular dilatation was the most common echocardiographic finding in COVID-19 patients [35]. Right ventricular dilation showed a significant association with serum levels of cardiac enzymes and was a significant predictor of mortality [35].

Cardiac magnetic resonance (CMR) imaging is the current gold-standard for characterizing cardiac morphology and function. Its exquisite soft-tissue resolution and non-invasiveness make it the imaging modality of choice to quantify ventricular volume and function. Multi-phase CMR allows for the detection and characterization of the different phases of myocardial inflammation and damage. The Lake Louise criteria is the combination of CMR features for diagnosing acute myocardial injury. Edema characterizing acute myocardial inflammation is best seen on T2-weighted imaging while contrast-enhanced CMR shows early gadolinium enhancement indicating hyperemia, and late gadolinium enhancement (LGE) indicating myocardial necrosis, fibrosis, and scarring [36]. Despite its obvious advantage when used to characterize cardiac function and morphology in COVID-19 patients, there are important barriers to its widespread application in the acute setting. These barriers include its incompatibility with metallic implants, longer scan duration, the risk associated with gadolinium administration, especially in patients with renal failure, and its relative high cost [37]. Some recent studies have shown cardiac abnormalities on CMR in patients who recovered from COVID-19. In the study by Puntmann and colleagues that included 100 patients who recovered from COVID-19 (median interval median COVID-19 positivity and CMR examination was 71 days, range = 64–92 days), 78 patients had had abnormal CMR findings, including at least one of raised myocardial native T1 (n = 73), raised myocardial T2 (n = 60), myocardial LGE (n = 32), or pericardial enhancement (n = 22) [38]. A similar high prevalence of cardiac abnormalities defined on CMR and associated impaired ventricular function have been reported by others suggesting residual or persisting cardiac changes induced by SARS-CoV-2 infection [39, 40].

Residual myocardial morphologic and functional changes on imaging may not be unexpected in individuals who recovered from severe COVID-19. Studies done in athletes who recovered from asymptomatic or mildly symptomatic COVID-19 have shown a high prevalence of morphologic and functional myocardial impairment in these athletes [41]. CMR appears to have the best sensitivity for detecting residual cardiac changes in athletes who recovered from COVID-19 even when serum levels of biomarkers of myocardial damage and other imaging testing are within normal limits [42]. Beyond the myocardium, the pericardium and pericardial space also show residual changes in the hearts of athletes who recovered from COVID-19. In the study by Brito and colleagues which recruited 54 consecutive student-athletes (16, 36, and 2 students were asymptomatic, mildly symptomatic, and moderately symptomatic for COVID-19, respectively) with a mean age of 19 years to undergo echocardiography and CMR for the assessment of post-COVID-19 cardiac changes [43]. Late pericardial enhancement and pericardial effusion were seen in 40% and 58% of athletes, respectively. The pericardial changes seen on echocardiography were less striking compared with CMR findings [43]. These findings support the conclusion that derangement in cardio-pericardial morphology and function occurs in young individuals with non-severe COVID-19 and may persist beyond the disease recovery. The long-term prognosis of these changes, however, remains to be determined.

## Vascular endothelial injury in COVID-19

Vascular endothelial changes are prevalent in SARS-CoV-2-infected patients. Blood vessels are present in all organs of the body. The deleterious vascular changes induced SARS-CoV-2 in patients with COVID-19 may suggest that vascular injury contributes to the multi-organ derangement seen in COVID-19. The vascular endothelium plays an important role in ensuring vascular relaxation, laminar blood flow, inhibition of platelet aggregation and coagulation, and fibrinolysis [44]. Vascular endothelial dysfunction, on the contrary, promotes vasoconstriction, turbulent blood flow, thrombus and atheroma formation, and impaired fibrinolysis [45].

**Fig. 3:**
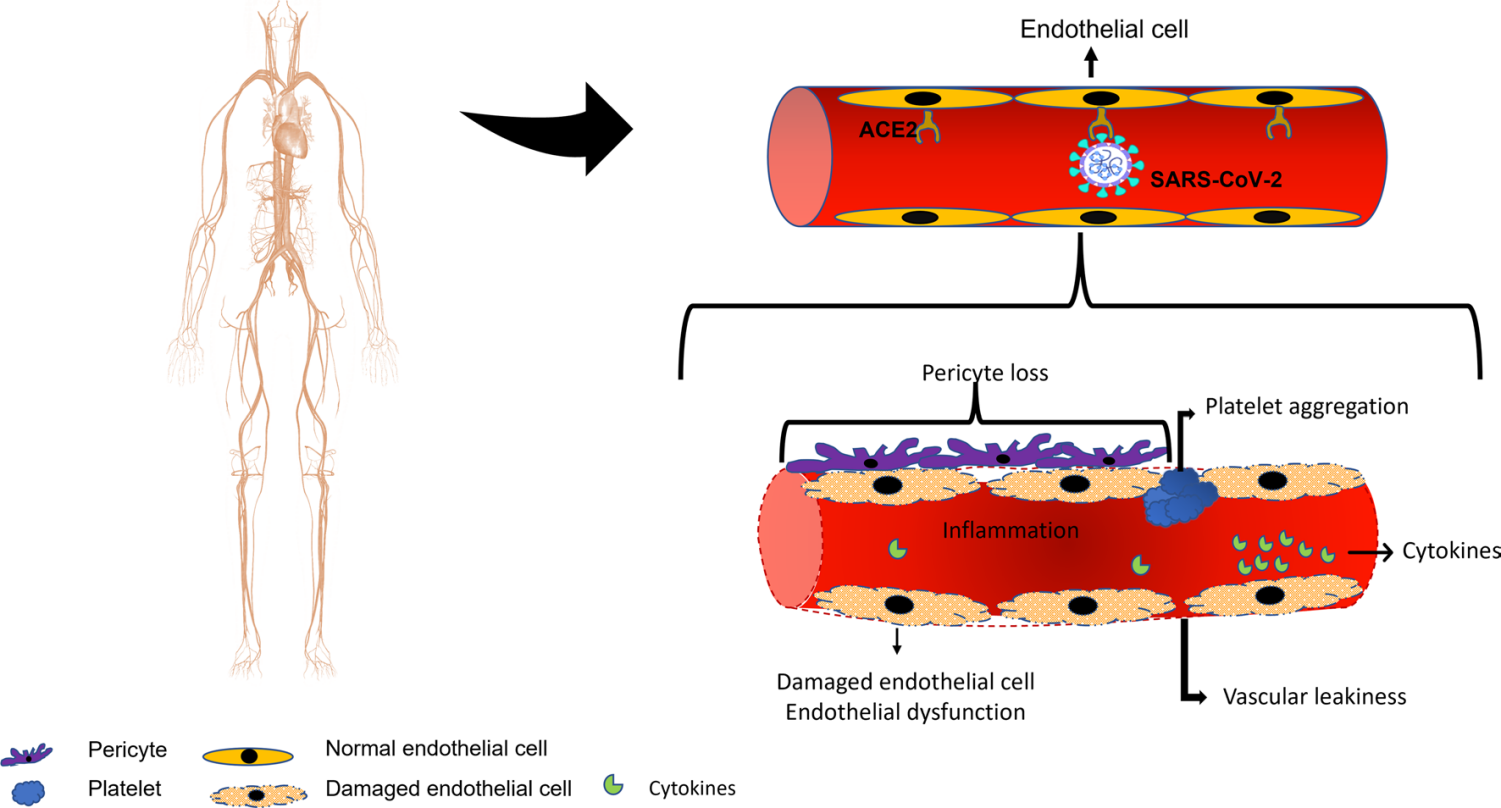
Schema showing the mechanisms of endothelial damage caused by SARS-CoV-2. These mechanisms include direct SARS-CoV-2 infection of the endothelial cells causing endothelial injury and vessel wall inflammation; down-regulation of ACE-2 caused by endothelial infection leading to vascular inflammation, thrombosis, increased vascularity, and microvascular dysfunction; vascular inflammation and endothelial injury induced by high circulating levels of cytokines; endothelial injury induced by high circulating levels of reactive oxygen species released by activated leucocytes; and SARS-CoV-2-induced pericytes loss leading to endothelial dysfunction and increased leakiness of the microvasculature. All these injuries contribute to hypercoagulability and increased intravascular clot formation

Angiotensin-converting enzyme-2 (ACE-2) works intimately with the Renin–Angiotensin–Aldosterone system (RAAS) to regulate cardiovascular function. The RAAS functions to maintain hemodynamic homeostasis through its regulation of water and solute balance in the body. Renin secreted from the juxtaglomerular cells of the kidney in response to low sodium content in the glomerular filtrate reaching the macula densa catalyses the conversion of the liver-produced angiotensinogen to angiotensin I. Angiotensin-converting enzyme (ACE) converts angiotensin I to angiotensin II. Angiotensin II is a potent vasoconstrictor and an inducer of vascular inflammation. The RAAS also plays regulatory roles in other organs such as heart, blood vessels, kidneys where it regulates local blood flow, cellular proliferation, and apoptosis [46]. ACE-2 that serves as receptor for SARS-CoV-2 is different from ACE of RAAS system in the substrate it acts on and the physiologic effects it elicits [47]. ACE-2 plays a balancing role in counteracting the vasoconstrictive and proinflammatory functions of the RAAS. ACE-2 acts on Mas receptor (MasR), a G protein-coupled receptor, leading to the conversion of angiotensin II to angiotensin 1–7 [48]. Angiotensin 1–7, via nitric oxide (NO), mediates vasodilation and inhibits platelet activation and aggregation [49, 50]. ACE-2 is expressed in the endothelium and vascular smooth muscles of arteries and veins [51]. Vascular expression of ACE-2 makes the vessel wall a potential target for infection by SARS-CoV-2 (Fig. 3). It has been shown that SARS-CoV-2 infects endothelial cells [52]. Endothelial infection by SARS-CoV-2 leads to vessel wall inflammation and endothelial cell death [53]. Also, SARS-CoV-2 infection leads to the down-regulation of ACE-2 expression with a consequent low level of Angiotensin 1–7 shifting the balance towards the proinflammatory and pro-thrombotic effect of angiotensin II. Down-regulation of ACE-2 may also activate the kallikrein-bradykinin pathway, leading to increased vascular permeability, tissue edema, and microcirculatory dysfunction [54].

The vascular endothelium is exposed to high circulating levels of proinflammatory cytokines associated with the cytokine storm that characterizes severe COVID-19. These high cytokine levels induce upregulation of endothelial cell adhesion molecules and chemokines, leading to leukocyte recruitment and inflammation in the vessel wall [55]. Again, this vessel wall inflammation causes endothelial cell death and a consequent increase in vascular permeability and end-organ damage. Reactive oxygen species (ROS) generated by the activated inflammatory cells also contribute to endothelial damage.

Pericytes are found in the basement membrane of the vessel wall and function to maintain the integrity of the endothelial layer of the microvasculature. Pericytes have been reported to express ACE-2 making them a potential target for infection by SARS-CoV-2 [56]. While infection of pericytes by SARS-CoV-2 remains to be proven, dysfunction of pericytes have been reported in patients with severe COVID-19 [57]. Loss of pericytes function disrupts the harmonious crosstalk between them and the endothelial cells promoting capillary endothelial dysfunction, which may be responsible for wall thickening of the venules and capillaries seen in COVID-19 patients [58, 59].

## Endothelial injury, coagulopathy, and COVID-19

Activation of the coagulation system is one of the consequences of endothelial injury. The coagulation system functions in three ways: (1) vascular changes and the formation of platelet plug, (2) formation of fibrin for clot stabilization, and (3) breakdown of the clot to restore microcirculation [60]. All the phases of the coagulation system are deranged in COVID-19 in favor of excessive thrombosis and inhibition of fibrinolysis. Endothelial injury causes platelet activation, its adherence to the sub-endothelial matrix, and the formation of platelet plug. Direct platelet activation by SARS-CoV-2 is possible via binding to platelet-expressed ACE-2, causing the release of clotting factors and pro-thrombotic factors [61]. This explains the increased level of platelet activation and platelet-monocyte aggregation formation that have been demonstrated in patients with severe COVID-19 compared with patients with mildly symptomatic or asymptomatic COVID-19 [62]. Widespread endothelial injury in COVID-19 causes the release of tissue factor that activates the coagulation cascade. The activation of cells of the monocyte/macrophage lineage by direct viral interaction also causes the release of tissue factor leading to the activation of the extrinsic component of the coagulation cascade [63]. Direct SARS-CoV-2 infection of neutrophils occurs, causing the production of neutrophil extracellular traps (NETs) that can activate factor XII triggering the activation of the intrinsic component of the coagulation cascade [64]. Prolonged immobilization, complement activation, and high levels of anti-phospholipid antibodies are other factors with pro-thrombotic properties that are prevalent among patients with severe COVID-19 [60, 65]. Plasminogen activator inhibitor 1 (PAI-1) is an inhibitor of the fibrinolytic system whose levels are elevated in patients with severe COVID-19 [66]. PAI-1 is contained within endothelial cells and platelets. Endothelial damage causes the release of PAI-1 leading to a state of hypofibrinolysis.

## Clinical manifestations of coagulation disorders in COVID-19

Vascular endothelial injury and other immunologic changes induced by SARS-CoV-2 infection predispose to arterial and venous thrombosis. A recent meta-analysis of 26 studies including 4382 patients that assessed the incidence, prognosis, and laboratory indicators of venous thromboembolism (VTE) in hospitalized patients with COVID-19 reported an incidence of 28.3% (95% CI: 21.6%–35.4%) for VTE [67]. The incidence of VTE among non-selected COVID-19 patients was 17.2% (95% CI: 11.4%–23.8%), indicating a lower but not insignificant risk of VTE among the general population of patients with COVID-19, including those with asymptomatic and mild forms of disease. Deep vein thrombosis (DVT) and pulmonary embolism (PE) represent the two extremes of the clinical manifestation of VTE. The pooled incidence of central and peripheral PE among hospitalized COVID-19 patients were 6.8% (95% CI: 1.8%–14.2%) and 12.3% (95% CI: 6.1%–20.2%), respectively. The incidence of PE was much higher among patients with severe COVID-19, seen in 21.7% (95% CI: 14.8–29.3) of patients. Patients with severe COVID-19 complicated by VTE were more likely to die compared with patients with severe COVID-19 not complicated by VTE (Odds ratio, OR = 2.02, 95% CI: 1.15–3.53). Development of VTE was significantly associated with elevated blood levels of D-dimer, low lymphocyte count, and longer prothrombin time, reflecting increased coagulation, an attempt at clot breakdown, and consumption of clotting factors [67]. Given the recognized heightened risk of VTE among hospitalized COVID-19 patients, anticoagulation therapy has now been included as part of the standard of care for these patients. In another meta-analysis that specifically looked at VTE incidence among critically ill COVID-19 patients receiving prophylactic or therapeutic anticoagulation therapy, the incidence of VTE remained high among this population of patients despite prophylaxis or therapy for VTE [68]. This finding calls for a continued vigilance for VTE in the care of patients with severe COVID even while on anticoagulation therapy.

Arterial thrombosis manifests clinically as myocardial infarction, stroke, and limb and visceral arterial ischemia. In a meta-analysis of 19 studies including 8249 patients, the pooled incidence of arterial thromboembolism (ATE) was 4.0% (95% CI: 2.0%–6.5%) [69]. The various forms of ATE occurred with incidence of 1.1% (95% CI: 0.2%–3.0%) for myocardial infarction/acute coronary syndrome, 1.6% (95% CI: 1.0%–2.2%) for ischaemic stroke, and 0.9% (95% CI: 0.5%–1.5%) for other forms of ATE [69]. In addition to a higher risk of ischaemic stroke in COVID-19 patients, SARS-COV-2-infected patients are more likely to develop severe disability from stroke or die from their disease when compared with patients with stroke but without SARS-CoV-2 infection [70].

## Radionuclide imaging of subclinical vascular endothelial injury

Vascular endothelial injury causing endothelial dysfunction is the primary pathophysiologic driver of the vascular complications of COVID-19. Endothelial injury is characterized by changes that can be targeted by radionuclide agents for non-invasive imaging. These changes include the expression of cell adhesion molecules by the vascular endothelium and the transmigration of activated inflammatory cells into the vessel intima [71]. These changes create an inflammatory milieu in the vessel wall. The utility of radionuclide agents for targeting vessel intima changes in endothelial injury has been recently reviewed by us and others [72, 73]. Radionuclide imaging by positron emission tomography (PET) provides a sensitive avenue for non-invasive imaging of in vivo biologic processes. The lower spatial resolution of the PET system is complemented by the anatomic imaging modalities like computed tomography (CT) or MRI to which it is interphase as hybrid PET/CT or PET/MR. Vascular inflammation is the precursor lesion of atherosclerotic cardiovascular diseases. PET/CT imaging with fluorodeoxyglucose (FDG), a glucose analog trapped by inflammatory cells, has been shown to be a valuable tool for the non-invasive assessment of vascular inflammation as the precursor lesion for atherosclerotic cardiovascular diseases [74]. Increased vascular uptake of FDG has been shown as a risk factor for atherosclerotic cardiovascular diseases in different conditions, including human immunodeficiency virus (HIV), chronic kidney disease, post-traumatic stress disorder, and rheumatological disorders [75–78].

The role of FDG PET/CT in assessing arterial inflammation as a consequence of endothelial injury has been evaluated in patients who recovered from COVID-19. In a prospectively pilot study comparing vascular FDG uptake as a marker of arterial inflammation in patients with least one persistent symptom 30 days or more after recovering from COVID-19 (the so-called long COVID) versus a control group of patients without COVID-19, Sollini and colleagues reported mild to moderate FDG uptake in the vessels and bone marrow of patients with long COVID compared with control in a qualitative analysis of images [79]. However, on quantitative analysis of vascular FDG uptake, the authors found no significant difference in the intensity of uptake between patients with long COVID and controls. In another study from the same group, while FDG uptake was prevalent among patients with long COVID on visual analysis of images, the quantified intensity of uptake was not significantly different between long COVID patients and control [80]. A lack of significantly increased vascular FDG uptake in COVID-19 patients compared with controls reported in these studies may be related to the modest number of patients included in the studies. Due to the small diameter of the major arteries in the human body, there is a significant photon intensity underestimation on PET imaging due to partial volume effect resulting from the limited spatial resolution of the PET system [81]. For this reason, certain imaging parameters must be observed during imaging of arterial FDG uptake to reduce the impact of photon underestimation on the quantified parameters [82, 83]. In the studies by Sollini and colleagues, these optimized parameters were not applied. They may have contributed to the lack of significant difference in the quantified vascular FDG uptake between long COVID patients and controls. Lack of standardization of PET imaging parameters is a known factor implicated in negative studies of vascular inflammation imaging [84].

Given the important role arterial inflammation plays in the future development of atherosclerotic cardiovascular diseases [85], more studies are necessary for the follow-up of COVID-19 survivors. PET imaging offers a suitable imaging modality for this indication since it can detect arterial inflammation, which is present at a much earlier timeline in atherogenesis before other morphological vascular changes detectable by anatomic imaging develop.

## Conclusions and future perspectives

SARS-CoV-2 is a respiratory virus that has caused significant morbidity and mortality since it was first identified as a cause of human infection in December 2019. Although it is acquired via the respiratory route, SARS-CoV-2 causes significant damage to the cardiovascular system, with cardiovascular disease manifestations responsible for a significant proportion of mortality due to it. Also, cardiovascular morbidity is a significant driver of unfavorable disease outcomes in COVID-19 patients. Therefore, it is important that managing physicians identify the cardiovascular disorders that precede COVID-19 and those induced by it to manage affected patients appropriately and adequately. The sensitive non-invasive opportunity afforded by radionuclide imaging with PET may be explored further in gaining insights into the molecular perturbations that occur in different organs contributing to an unfavorable outcome in COVID-19. PET techniques may also be applied in studying the long-term complications of COVID-19, especially in patients who experience persisting symptoms.

## Data Availability

Not applicable.

## Abbreviations

ACE-2: Angiotensin-converting enzyme-2
ARDS: Acute respiratory distress syndrome
ATE: Arterial thromboembolism
CMR: Cardiac magnetic resonance
COVID-19: Coronavirus disease 2019
CRS: Cytokine release syndrome
CT: Computed tomography
DNA: Deoxyribonucleic acid
DVT: Deep vein thrombosis
FDG: Fluorodeoxyglucose
LGE: Late gadolinium enhancement
MRI: Magnetic resonance imaging
PE: Pulmonary embolism
PET: Positron emission tomography
RAAS: Renin angiotensin aldosterone system
ROS: Reactive oxygen species
SARS-CoV-2: Severe acute respiratory syndrome coronavirus-2
TMPRSS2: Transmembrane serine protease 2
TnT: Troponin T
VTE: Venous thromboembolism
